# Influence of SS316L Nanoparticles on the Sintered Properties of Two-Component Micro-Powder Injection Moulded Bimodal SS316L/Zirconia Bi-Materials

**DOI:** 10.3390/ma17225536

**Published:** 2024-11-13

**Authors:** Al Basir, Abu Bakar Sulong, Norhamidi Muhamad, Afifah Z. Juri, Nashrah Hani Jamadon, Farhana Mohd Foudzi, Nabilah Afiqah Mohd Radzuan, Kambiz Rashidi

**Affiliations:** Department of Mechanical and Manufacturing Engineering, Faculty of Engineering and Built Environment, Universiti Kebangsaan Malaysia, Bangi 43600, Selangor, Malaysia; al.basir005@yahoo.com (A.B.); norhamidi@ukm.edu.my (N.M.); afifahjuri@ukm.edu.my (A.Z.J.); nashrahhani@ukm.edu.my (N.H.J.); farhana.foudzi@ukm.edu.my (F.M.F.); afiqah@ukm.edu.my (N.A.M.R.); kambiz.rashidi66@gmail.com (K.R.)

**Keywords:** nanoparticles, feedstocks, bimodal SS316L/3YSZ micro-components, two-component micro-powder injection moulding, sintering

## Abstract

Two-component micro-powder injection moulding (2C-μPIM) is a prospective approach for fabricating bi-material micro-components of stainless steel 316L (SS316L) and 3 mol% yttria-stabilised zirconia (3YSZ) at an appealing cost. However, the fundamental challenge lies in preventing the formation of large-scale cracks at the interface of two different materials during sintering. This study investigated how SS316L nanoparticles in bimodally configured SS316L powder that incorporated both nanoparticles and microparticles influenced the sintering of 2C-μPIM-processed miniature bi-materials made of bimodal SS316L and 3YSZ. In this study, feedstocks were developed by integrating monomodal (micro-sized) SS316L powder, three types of nano/micro-bimodal SS316L powders, and 3YSZ powder individually with palm stearin and low-density polyethylene binders. The results indicated that increasing the SS316L nanoparticle content to 45 vol.% caused a 19.5% increase in the critical powder loading in the bimodal SS316L powder as compared to that in the monomodal SS316L powder. The addition of SS316L nanoparticles increased the relative density and hardness of the sintered bi-materials, with the maximum values obtained being 96.8% and 1156.8 HV, respectively. Field emission scanning electron microscopy investigations revealed that adding 15 vol.% and 30 vol.% SS316L nanoparticle contents reduced interface cracks in bi-materials significantly, while 45 vol.% resulted in a crack-free interface.

## 1. Introduction

Over the last few decades, industrial developments have increased the need for the mass production of complex-shaped components with small dimensions or micro-sized components [1,2,3]. A version of the powder injection moulding (PIM) technique, the micro-powder injection moulding (µPIM) process presents an appealing commercial approach for the production of such micro-components [4,5]. The emergence of two-component micro-powder injection moulding (2C-μPIM) technology from µPIM demonstrates the fascination of the worldwide market for downsized products as well as the broad spectrum of functions that micro-components can possess. The ability to integrate two different materials into a single micro-sized component using 2C-μPIM is a compelling manufacturing strategy because of its economically feasible production approach and large range of material alternatives [6,7,8]. Several investigators have conducted the prototyping of various bi-material micro-components using 2C-μPIM. For instance, Imgrund et al. [7] produced a magnetic–non-magnetic micro-bi-material made from stainless steel 316L (SS316L) and stainless steel 17-4PH (SS17-4PH) for use in various micro-applications. Ruh et al. [8] fabricated a bi-material shaft-to-collar connection at the micro-level by using alumina (Al_2_O_3_) and zirconia (ZrO_2_) for medical devices. Piotter et al. [9] reported the development of a micro-sized bi-material heater (u-shaped) using titanium nitride (TiN) and Al_2_O_3_ for different engineering applications. Basically, 2C-μPIM undergoes the same processing steps as PIM and μPIM, which include mixing, injection moulding, debinding, and sintering, as is widely known. The 2C-μPIM process generally initiates with the generation of feedstocks, which involves blending two different types of powders with a multi-component binder system individually. The process of micro-injection moulding yields green bi-materials by using the previously generated feedstocks and either a sequential or a simultaneous mechanism. This step is followed by the debinding process through which the binder system is extracted from the green parts. The debound components are sintered in the last step, resulting in finished pieces with satisfactory density and final properties.

While producing via 2C-μPIM, it is challenging to completely wipe out the emergence of deformation and defects in the bi-material micro-components. The development of cracks, delamination, and pore bands at the joining region of 2C-μPIM-processed micro-sized bi-materials was reported in prior studies [8,10,11]. Making meticulous powder selections with an appropriate particle size distribution is a major step in mitigating the aforementioned flaws. As a general rule in μPIM, the mean particle size of the powders should be at least ten times smaller than that of the minimum feature size [12]. This perspective stipulates that to produce bi-materials, the 2C-μPIM technique should use ultra-fine or nano-sized powder particles to obtain components with excellent dimensional stability, a better surface finish, and adequate physical and mechanical properties [13,14,15]. However, the large specific surface area of nano-sized powder particles decreases the powder loadings in the feedstocks dramatically and fosters challenges by significantly increasing shrinkage in the bi-materials during sintering, which eventually leads to the development of cracks at the joining region and sometimes inadequate joining or joining failure in the micro-components [16]. Moreover, the large specific surface area leads to increased interparticle friction. This significantly increases the viscosity of feedstocks and complicates the injection moulding procedure [13,17]. Furthermore, the exceedingly high cost of nanopowders is considered another significant drawback [18]. An approach towards overcoming such constraints in the 2C-μPIM process is to use a nano/micro-bimodal powder (N/M-BP) system. Mixing a nano-sized powder with a micro-sized powder with the same theoretical density leads to the development of N/M-BP. This approach preserves the advantageous attributes of nanopowders while minimising their drawbacks [19].

Rajabi et al. [12] studied the shrinkage behaviour and microstructural evolution of μPIM-processed SS316L parts prepared using N/M-BPs. In contrast, Liu et al. [20] conducted research on the mouldability of zirconia micro-gears and found that the μPIM technique could effectively construct 200-μm micro-gears. In the same vein, Hanemann et al. [21] studied the properties of feedstocks to produce defect-free zirconia micro-parts through μPIM and reported that the feedstock containing stearic acid exhibited the best flow behaviour and homogeneity. While investigations have been performed to fabricate bimodally configured SS316L and zirconia micro-components by using the μPIM process, there are glaringly insufficient reports demonstrating the sintering behaviour of metal/ceramic bi-material micro-components of nano/micro-bimodal SS316L and zirconia produced using 2C-μPIM.

In this research work, nano-sized and micro-sized SS316L and 3 mol% yttria-stabilised zirconia (3YSZ) were chosen as the study materials. SS316L has many advantages, such as high corrosion resistance (pitting resistance equivalent number (PREN) is between 22.6 and 29.5), ease of production, and excellent mechanical strength (tensile strength ranging from 430 MPa to 530 MPa, with a yield strength ranging from 140 MPa to 350 MPa) [22,23]. 3YSZ is an extensively used ceramic with a large number of beneficial properties, such as superior flexural strength (900 MPa to 1200 MPa) and fracture toughness (4 MPa√m to 8 MPa√m), high biocompatibility, and good thermal stability [24,25,26]. Applications ranging from the electrical, biomedical, aerospace, and automotive sectors could considerably benefit from the micro-level integration of bimodal SS316L and 3YSZ. The primary objective of the current study was to investigate the effects of the addition of SS316L nanoparticles to powder–binder blends on the sintering characteristics of bimodal SS316L/3YSZ micro-components fabricated using 2C-μPIM.

## 2. Materials and Methods

### 2.1. Materials

**Figure 1:**
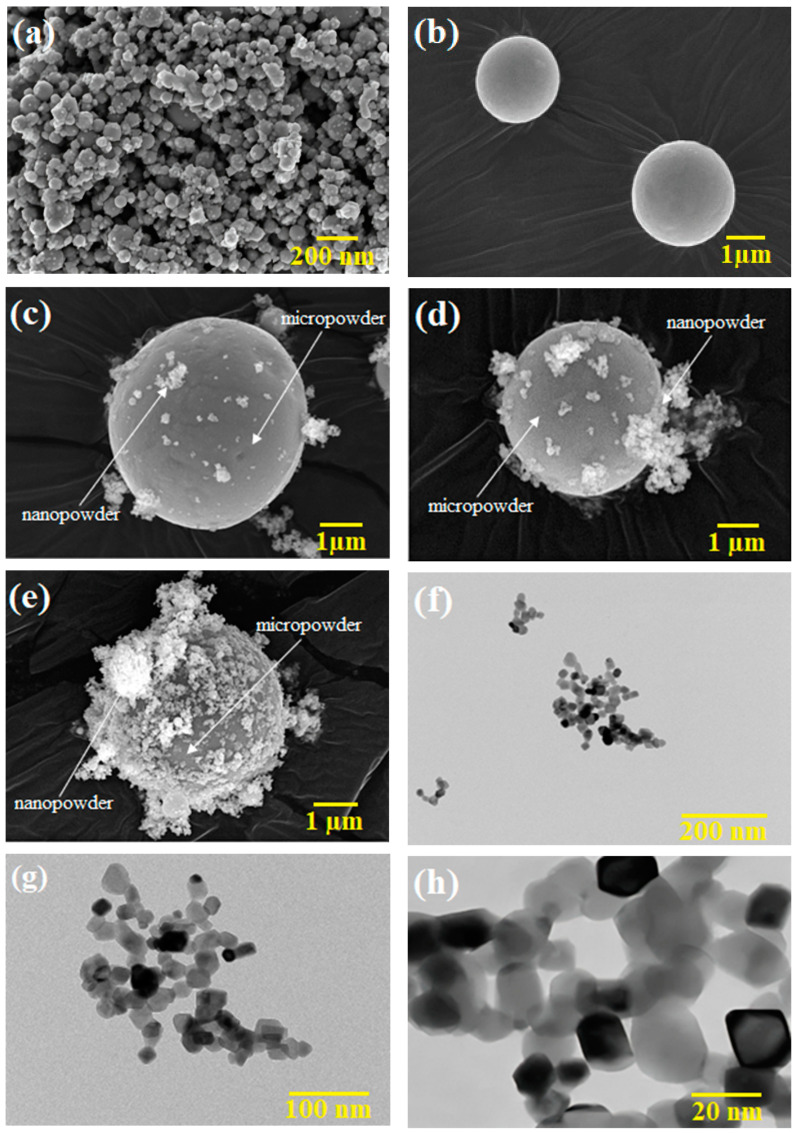
Morphology of the powders: (**a**) FESEM image of SS316L nanopowder, (**b**) FESEM image of SS316L micropowder, (**c**) FESEM image of bimodal SS316L powder with nanopowder content of 15 vol.%, (**d**) FESEM image of bimodal SS316L powder with nanopowder content of 30 vol.%, (**e**) FESEM image of bimodal SS316L powder with nanopowder content of 45 vol.%, and (**f**–**h**) TEM images of 3YSZ powder at different magnifications.

The metal and ceramic powders used in this study were SS316L and 3YSZ, respectively. In this study, nano- and micro-sized SS 316L powders with average sizes of 150 nm and 7 µm were supplied by Hongwu Nanometer Co. Ltd., Guangzhou, China, and Epson Atmix Corporation, Aomori, Japan, respectively. 3YSZ, with an average size of 30 nm reported by the supplier, was purchased from Inframat Advanced Materials LLC, Manchester, CT, USA. The two different sizes of SS316L powders were blended using a Fritsch Pulverisette-6 (Idar-Oberstein, Germany) planetary mono mill for 3 h to formulate N/M-BPs. The prepared materials were monomodal (micro-sized) SS 316L powder and three N/M-BPs of SS316L with nanoparticle contents of 15 vol.%, 30 vol.%, and 45 vol.%. Such SS316L powders were designated as micropowder, 15:85 N/M-BP, 30:70 N/M-BP, and 45:55 N/M-BP, respectively. The average particle sizes of bimodal SS316L powders with nanoparticle contents of 15 vol.%, 30 vol.%, and 45 vol.% were 6.37 µm, 6.26 µm, and 5.88 µm, respectively. The particle sizes of nano- and micro-sized powders were measured using Malvern Zeta Sizer and Malvern Mastersizer 2000 (Malvern, UK), respectively. AccuPyc II 1340 Pycnometer was used to assess the pycnometer densities of the metal and ceramic powders. The pycnometer densities of SS316L powders relating to the micropowder, 15:85 N/M-BP, 30:70 N/M-BP, and 45:55 N/M-BP were 7.74 g/cm^3^, 7.69 g/cm^3^, 7.62 g/cm^3^, and 7.53 g/cm^3^, respectively. In contrast, the pycnometer density for the 3YSZ powder was 6.03 g/cm^3^. The morphologies of the metal and ceramic powders (Figure 1a–h) were observed using a field emission scanning electron microscope (FESEM, Zeiss Merlin Compact, Jena, Germany) and a transmission electron microscope (TEM, Talos L120C, Waltham, MA, USA), respectively. Figure 1c–e reveals an apparent bimodal arrangement of the SS316L powder. The binder system and composition were chosen on the basis of prior research [27], with palm stearin used to enhance the wettability and flow properties and LDPE holding the powder particles together and providing sufficient strength for each micro-sized bi-material until sintering. When fabricating micro-components with a nano-sized powder, the binder is of particular significance as the nano-sized powder causes high viscosity and makes it difficult to fill small mould cavities [28]. The melting point and the degradation temperature of the binders used in the feedstocks were ascertained using a differential scanning calorimetry (DSC) analysis and a thermogravimetric (TGA) analysis, performed using NETZSCH DSC 214 Polyma and the NETZSCH Simultaneous Thermal Analyser (STA) 449 F3 Jupiter (Selb, Germany). Using the DSC and TGA data as the benchmarks, the temperature during the mixing and debinding procedures was determined [29,30,31]. The characteristics of the binders are demonstrated in Table 1.

### 2.2. Two-Component Micro-Injection Moulding (Green Part Preparation)

In the 2C-µPIM process, the final product quality is substantially affected by the content of the powder in the feedstock. As a result, it is important to prepare the feedstock by using the optimal powder loading; this quantity is established using the critical solid loading, which is the state where particles are packed as closely as possible despite the requirement for pressure from outside and all of the space between them is completely occupied with the binder [17,32]. The use of powder loading higher than the critical content increases the viscosity of the feedstock enormously by lowering the binder content. In this study, a critical powder volume concentration (CPVC) analysis was conducted to establish the critical powder loadings of each powder by using the oil absorption technique in accordance with ASTM standard 281–31 [33]. The optimal powder loadings were determined to be 2 vol.% less than the critical powder loadings to provide flexibility in the process [17]. With the use of a Brabender W50 EHT internal mixer, the feedstocks were prepared by mixing the optimal amounts of powders with the binder system, while maintaining a mixing temperature of 150 °C and a consistent speed of 30 rpm. The viscosity of the feedstocks was measured at three different temperatures between 190 °C and 230 °C by using a Shimadzu CFT-500D (Kyoto, Janpan) capillary rheometer with a diameter of 1 mm and 10 mm long die. To acquire the rheological data, a specified quantity of feedstock was introduced into the capillary barrel, which was subsequently preheated for a duration of 2 min prior to the initiation of the test. These data were useful in predicting the flow of the feedstocks into the mould cavity.

Green bi-material micro-components in the form of dumbbells (9 mm in length and 0.9 mm in thickness) were produced using an injection moulding machine (DSM Xplore) utilising the produced feedstocks. In this experiment, a sequential mechanism based on previous research [34] was used to produce bi-materials, which eventually formed a bond between the metal and the ceramic via the injection moulding process. Table 2 lists the parameters used in the production of the green micro-sized bi-materials.

### 2.3. Debinding (Brown Part Preparation)

**Figure 2:**
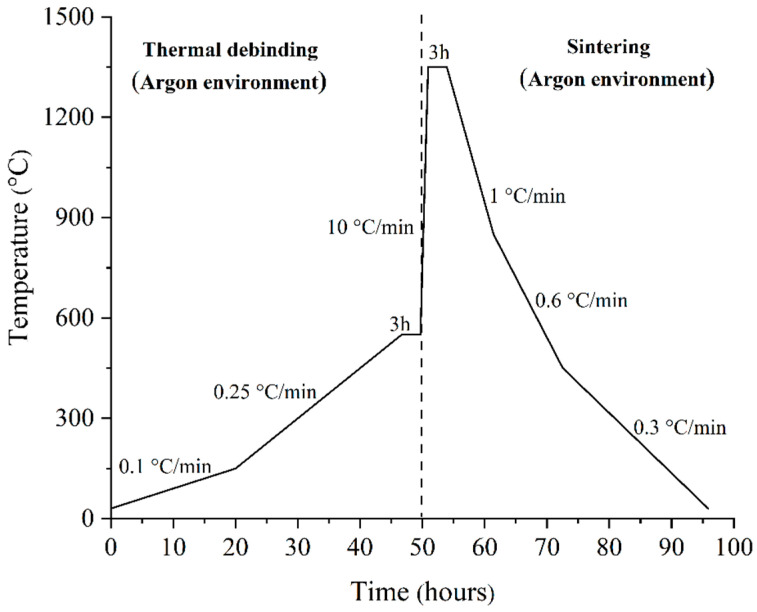
Diagram depicting the steps involved in thermal debinding and sintering.

The brown bi-material is usually produced by the removal of the binders from the green bi-material during the debinding process. Debinding reduces the backbone binder and escalates porosity, making bi-materials susceptible to a joining failure. The use of a nano-sized powder further complicates the procedure by lowering the interparticle gaps [35]. Upon the consideration of such challenges, a combination of solvent and thermal debinding techniques was used in this experiment to achieve defect-free brown samples. The solvent debinding process was carried out using a BINDER FDL 115 safety drying oven to remove the soluble binder (palm stearin). The tests were conducted at four different temperatures, ranging from 40 °C to 70 °C. The samples were submerged in acetone for 40 min, and the weight change following drying was used to determine the remaining palm stearin binder content. The thermal debinding process was conducted using a tube furnace (HTF-15/200–60) under argon gas. In this stage, the insoluble binder (LDPE) and the residual palm stearin were removed from the solvent debound samples in two phases. In the initial phase, samples were heated at a rate of 0.1 °C/min from 30 °C to 150 °C, while in the later phase, they were heated to 550 °C at 0.25 °C/min for 3 h. Figure 2 illustrates the diagram of the thermal debinding cycle.

### 2.4. Sintering

During the sintering process, all of the thermal debound samples were heated from 550 °C to 1350 °C at 10 °C/min for 3 h by using a tube furnace and atmosphere similar to that used during the thermal debinding process, as shown in Figure 2. The earlier studies by Song and Evans [36] and Meng et al. [37] were taken into account for choosing the sintering temperature to achieve optimum sintered properties. The cooling of the sintered samples was carried out in three phases, as shown in Figure 2. All of the samples were cooled at the cooling rates of 1 °C/min, 0.6 °C/min, and 0.3 °C/min, respectively, from 1350 °C to 850 °C, 450 °C, and 30 °C.

### 2.5. Characterisation of Micro-Sized Bi-Materials

With the use of the Archimedes approach and the MPIF Standard 42 as the baseline, the densities of the sintered bi-materials were measured. The linear shrinkage percentage of the samples based on MPIF standard 44 was determined by measuring the length differences in the samples pre- and post-sintering. The density and shrinkage measurements were carried out eight times for each nanopowder content. The polished surfaces of the samples were observed using FESEM. An energy dispersive X-ray elemental analysis (EDX) was used to assess the elements existing at the metal/ceramic interface of bi-materials. The hardness of the bonding region of the samples was tested using a HIGHWOOD hardness tester in accordance with MPIF standard 51. For the hardness measurement, samples were subjected to a 1 N load for 15 s. Each nanopowder content underwent five repetitions of the hardness test.

## 3. Results and Discussion

### 3.1. Critical Powder Loadings

**Figure 3:**
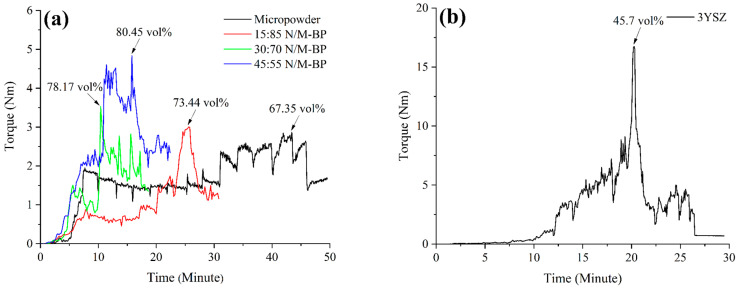
Critical powder contents of (**a**) monomodal and bimodal SS316L powders and (**b**) 3YSZ powder.

Though high powder loading improves the physical and mechanical properties of the final products, using very high powder loading not only complicates the process of mixing the powder and the binder but also increases feedstock viscosity because of the high interparticle friction, resulting in an inadequate amount of feedstock to flow into the cavity of the mould during the injection moulding stage [38]. Therefore, it is preferable to use optimal powder loading in the 2C-µPIM process. When optimal powder loading is used, sintered components have fewer defects overall and have superior physical and mechanical properties as a result [39]. The optimal solid loading, which depends on the critical powder loading, must be determined prior to the feedstock preparation. The critical value of the volume fraction is determined by the ratio of the powder to the binder, which also plays a major role in determining the effectiveness of the later processes. The critical powder loading data of the metal (monomodal and bimodal SS316L) and ceramic (3YSZ) powders are illustrated in Figure 3. As shown in Figure 3a, upon the addition of the nano-sized SS316L powders (15 vol.%, 30 vol.%, and 45 vol.%) to the micro-sized SS316L powder, the maximum torque value increased from 2.82 Nm to the range of 3.01 Nm–4.84 Nm. A greater level of friction between the nanoparticles and the microparticles could have caused this [12]. According to Figure 3a, the critical powder loadings of the micropowder, 15:85 N/M-BP, 30:70 N/M-BP, and 45:55 N/M-BP were 67.35 vol.%, 73.44 vol.%, 78.17 vol.%, and 80.45 vol.%, respectively. These data demonstrate a 9.04–19.45% increase in the critical powder loadings in bimodally configured SS316L powders relative to the monomodal SS316L powder. A prior study [12] that observed the inclusion of nano-sized SS316L powders with different contents increased the critical powder loadings in the nano/micro-bimodal SS316L powder in comparison to the monomodal SS316L powder validated this finding. This implies that the broad particle size distributions or bimodally configured nano/micro distributions are preferable for enhancing the loading of the powder because of the nanoparticles’ ability to occupy the interstitial gaps between the microparticles [17]. Moreover, as shown in Figure 3b, the highest torque displayed by the 3YSZ powder was 16.74 Nm, while a critical powder loading of 45.70% was achieved. The critical value obtained for 3YSZ in this research was approved by a previous report [40]. A comparison of Figure 3a with Figure 3b revealed that the critical value of the 3YSZ powder was 32.15% lower than that of the monomodal SS316L powder, and it was considerably lower in the range of 37.77% to 43.19% than that of the bimodal SS316L powders. This could be attributed to the fact that the 3YSZ nanopowder had a larger surface area and therefore needed greater amounts of the binder (oil) to entirely coat each particle [41]. On the basis of the critical values, the optimal powder loadings were selected for process flexibility. For SS316L, the optimal powder loadings for the micropowder, 15:85 N/M-BP, 30:70 N/M-BP, and 45:55 N/M-BP were 65 vol.%, 71 vol.%, 76 vol.%, and 78 vol.%, respectively. In contrast, the optimal powder loading for 3YSZ was 44 vol.%.

### 3.2. Mixing of Powders and Binders

**Figure 4:**
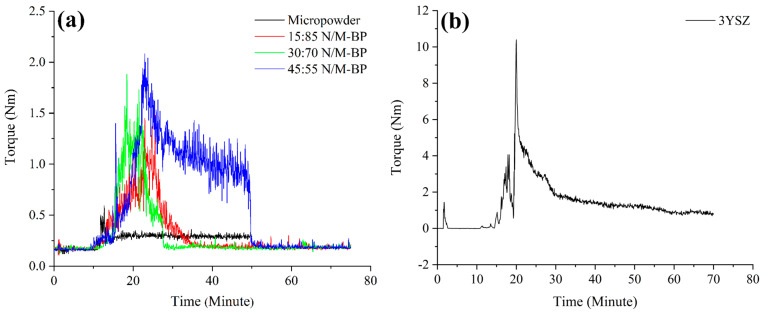
Mixing curves of the feedstocks: (**a**) monomodal and bimodal SS316L and (**b**) 3YSZ.

**Figure 5:**
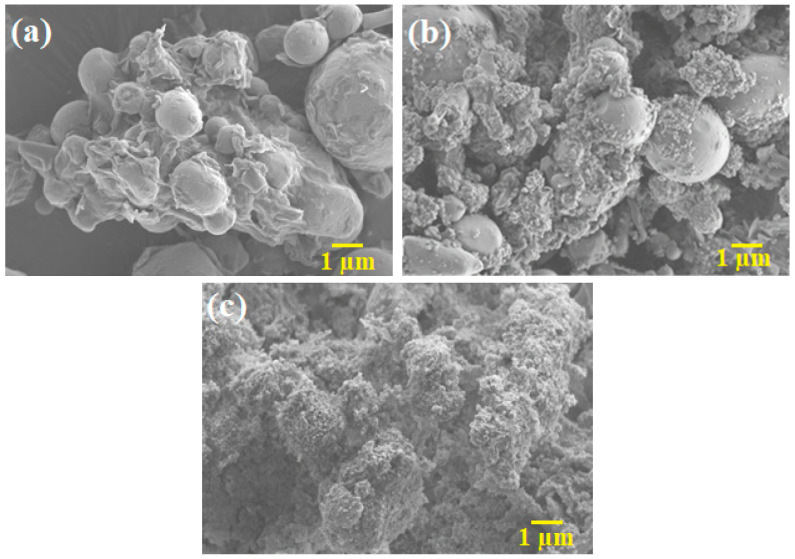
FESEM micrographs of the feedstocks: (**a**) monomodal SS316L, (**b**) 45:55 bimodal SS316L, and (**c**) 3YSZ.

The formulation of the feedstocks of monomodal SS316L, bimodal SS316L, and 3YSZ was conducted on the basis of weight by using the theoretical densities of their ingredients. To obtain balanced powder–binder feedstocks, an assessment of the weight fraction of the powders and the binders was conducted. The applied mixing temperature (150 °C) was not only lower than the temperature at which the palm stearin binder began to decompose but also exceeded the melting temperature of the LDPE binder. Choosing an appropriate mixing temperature makes it easier to melt the binders entirely while also preventing them from degrading. The mixer that correlated the measurement of the mixing torque with time was used to evaluate the homogeneity of the feedstocks. Figure 4 illustrates the mixing curves of the feedstocks, which were obtained because of the mixing of the optimal amounts of powders with the binder system. According to Figure 4a, the torque values increased significantly in the bimodal SS316L feedstocks containing 15 vol.%, 30 vol.%, and 45 vol.% nano-sized SS316L powder particles when compared with the monomodal SS316L feedstock. The reason for this could be that during the preliminary stage of mixing, a greater torque was needed to lessen the agglomerated clusters that were present in the nano-sized SS316L powder particles. A comparison of Figure 4a,b revealed that the 3YSZ feedstock demonstrated a substantially greater torque during the beginning stage of the mixing process than the bimodal SS316L feedstocks. This could be attributed to the fact that the 3YSZ powders were substantially smaller in size than the bimodal SS316L powders, resulting in more agglomerated clusters of the 3YSZ powder particles, which required a significantly higher toque to decrease. According to Figure 4, the homogeneity of the metal- and ceramic-based feedstocks was confirmed by the attainment of the steady value following an early augmentation of the mixing torque with time. Similar behaviour was observed in previous studies [21,42] during the preparation of feedstocks using SS17-4PH and zirconia powders. In essence, a homogeneous feedstock improves the physical and mechanical properties of the sintered part in addition to providing one free of defects [43,44]. The FESEM micrographs of the monomodal SS316L, 45:55 bimodal SS316L, and 3YSZ feedstocks are shown in Figure 5. According to Figure 5, while the SS316L powder particles were reasonably wrapped with binders, the 3YSZ powder particles were fully enveloped in binders.

### 3.3. Rheology

**Figure 6:**
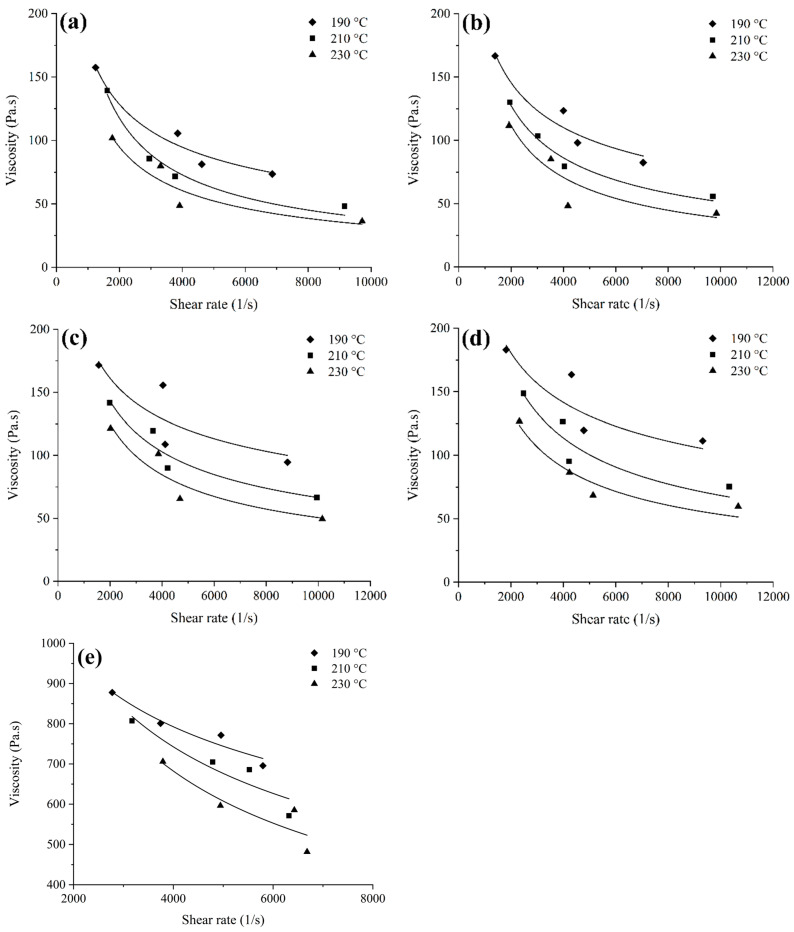
Variation in viscosity with shear rate for (**a**) monomodal SS316L, (**b**) 15:85 bimodal SS316L, (**c**) 30:70 bimodal SS316L, (**d**) 45:55 bimodal SS316L, and (**e**) 3YSZ feedstocks.

To forecast the feedstock flow behaviour and assess the mould filling throughout the injection moulding process, a rheological analysis is considered an essential technique. The assessment of the rheological properties in the present study was carried out in compliance with the profiling of the viscosity of the metallic and ceramic feedstocks on the grounds of temperature against shear rate. Figure 6 illustrates the viscosity versus shear rate graph of the prepared feedstocks at 190 °C, 210 °C, and 230 °C. Figure 6 illustrates that an increasing shear rate reduces the viscosity of all of the feedstocks, indicating pseudo-plastic behaviour or shear thinning. There have been reports of such phenomena in the literature [27,45]. The capacity of the injection-moulded object to maintain its shape is largely streamlined by pseudo-plastic activity, which also ensures that the mould cavity is filled effectively [17]. Figure 6 demonstrates the viscosity ranges of monomodal SS316L, 15:85 bimodal SS316L, 30:70 bimodal SS316L, 45:55 bimodal SS316L, and 3YSZ feedstocks, which were 73.51 Pa·s–157.45 Pa·s, 82.41 Pa·s–166.70 Pa·s, 94.50 Pa·s–171.57 Pa·s, 111.3 Pa·s–183.14 Pa·s, and 695.57 Pa·s–877.84 Pa·s, respectively, at 190 °C. These values decreased to 37.20 Pa·s–101.70 Pa·s, 42.40 Pa·s–111.31 Pa·s, 49.50 Pa·s–121.46 Pa·s, 59.70 Pa·s–126.80 Pa·s, and 481.76 Pa·s–705.91 Pa·s at 230 °C. The primary cause of this phenomenon was the decrease in affinity between the molecules of the multi-component binder system as the temperature increased [46]. Previous studies [47,48] have indicated that a viscosity level lower than 1000 Pa·s and a shear rate ranging from 10^2^–10^5^ s^−1^ can be used to enable an efficient feedstock flow to the mould cavity. The 15:85 bimodal, 30:70 bimodal, and 45:55 bimodal SS316L feedstocks showed 9.45–29.66%, 19.43–33.06%, and 24.68–60.48% higher viscosity, respectively, than the monomodal SS316L feedstock at 230 °C, according to a comparison of Figure 6a and Figure 6b–d. This can be attributed to the large specific surface area of nanoparticles, which resulted in increased interparticle friction and greater viscosities in the bimodal SSS316L feedstocks than in the monomodal SS316L feedstock. According to Figure 6, the significantly higher viscosity of the 3YSZ feedstock than that of the other metallic feedstocks could be attributed to the considerably lower powder particle size of the 3YSZ than that of the monomodal and bimodal SS316L powders.

The association between viscosity $\left(\eta \right)$ and shear rate $\left(\Upsilon \right)$ can be expressed by the power law as the monomodal 316L, bimodal 316L, and 3YSZ feedstocks demonstrated pseudoplastic behaviour:

(1) $$\eta =K\Upsilon^{n-1}$$

where K and n indicate the constant and the flow behaviour index, respectively. A value of n typically represents shear sensitivity, whereas the pseudo-plastic behaviour correlates to n<1. In accordance with Figure 6, Table 3 provides the calculated n values for the metallic and ceramic feedstocks. It is evident that as the temperature increased, the values of n decreased. The rheological properties of the feedstock under shear were affected by the interactions of the molten binder and powder particles. The value of n gradually decreased as the temperature of the metal- or ceramic-based feedstock increased as a consequence of the elevated mobility of the powder particles [49,50]. A significantly lower value of n indicated that the feedstock exhibited more pseudo-plastic behaviour. This was preferred for injection moulding as the feedstock viscosity had to immediately reduce as the shear rate increased at this stage [51,52]. Table 3 shows that the lowest n values for the monomodal SS316L, bimodal SS316L, and 3YSZ feedstocks were observed at 230 °C; therefore, this value was chosen as the melting temperature during the injection moulding step.

The temperature dependence of viscosity is another significant aspect to consider when examining the flow properties of a feedstock. The Arrhenius equation, which can be expressed as follows, is frequently utilised to clarify the association between feedstock viscosity and temperature:

(2) $$\eta \left(T\right)=\eta_{o\;}\exp\;\left(E/RT\right)$$

where $\eta_{o}$, E, R, and T represent the reference viscosity, flow activation energy, gas constant, and absolute temperature, respectively. This study used the slope of ln(η) vs. 1/T graph to determine E, which is a comparable approach to earlier research [18], and the findings are summarised in Table 4. Table 4 shows that the bimodal SS316L feedstocks exhibited a significantly greater E than the monomodal SS316L feedstock. In the case of the bimodal SS316L feedstocks, E increased as the amount of the SS316L nano-sized powder particles increased, with the 45:55 bimodal SS316L feedstock yielding the greatest value. In contrast, the 3YSZ feedstock demonstrated a higher E value than the monomodal SS316L feedstock; however, it was substantially lower than the values of the bimodal SS316L feedstocks. In essence, the greater value of E indicated that the feedstock viscosity was more sensitive to temperature, which increased the likelihood that the feedstock would solidify quickly during the injection moulding process [53]. In addition to facilitating the feedstock flow to the mould prior to hardening, a lower value of E reduced the defects in the components [48].

### 3.4. Injection Moulding

On the basis of rheological investigations, defect-free monomodal SS316L/3YSZ, 15:85 bimodal SS316L/3YSZ, 30:70 bimodal SS316L/3YSZ, and 45:55 bimodal SS316L/3YSZ bi-material micro-components were fabricated using the prepared metallic and ceramic feedstocks during the injection moulding stage. The utilisation of a mould temperature of 100 °C revealed the flowability of the monomodal SS316L feedstock into the mould cavity; however, the inadequate flowability of the bimodal SS316L feedstocks was commonly exhibited when the mould temperature was lower than 140 °C. According to previous investigations [17,46], the underlying reason for this occurrence is that during micro-injection moulding, the minute dimensions of the mould cavity lead the bimodal SS316L feedstocks to cool instantaneously, impeding the flowability of feedstocks at mould temperatures below 140 °C. In this study, a mould temperature of 140 °C was eventually selected to ensure the flowability of both the monomodal and the bimodal SS316L feedstocks. To prevent the development of flash defects in the injection-moulded bi-materials, an injection pressure of higher than 12 bar was not used. In general, insufficient injection pressure prevents sufficient feedstock from flowing into the mould cavity.

**Figure 7:**
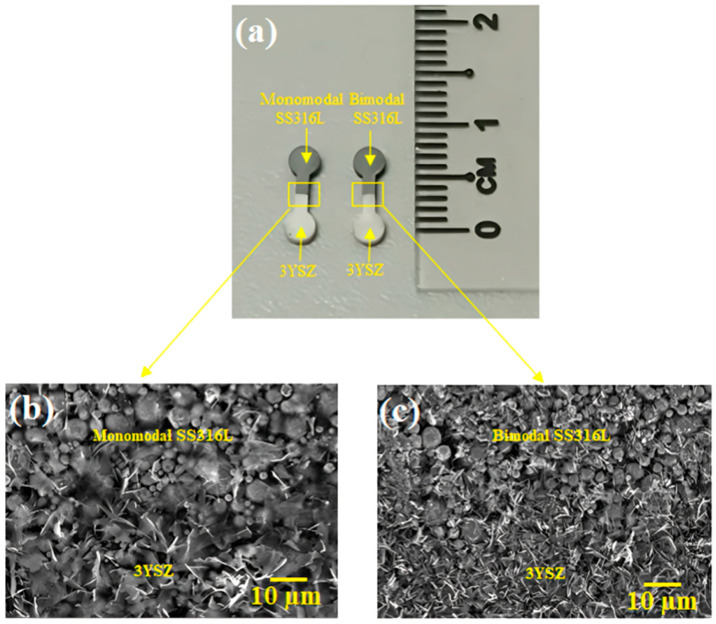
(**a**) Green monomodal SS316L/3YSZ and 45:55 bimodal SS316L/3YSZ micro-components, (**b**) FESEM image of the joining region of green monomodal SS316L/3YSZ micro-component, and (**c**) FESEM image of the joining region of green 45:55 bimodal SS316L/3YSZ micro-component.

The mould needs to be appropriately cooled down after injection to ensure that the bi-materials reach the necessary strength before demoulding the components. Demoulding was carried out carefully in this research work so as not to cause defects in any green components. Figure 7a depicts two-component micro-powder injection-moulded green monomodal SS316L/3YSZ and 45:55 bimodal SS316L/3YSZ micro-components that were free of any defects such as cracking, flashing, or jetting. The FESEM images of the joining region of the bi-materials are shown in Figure 7b,c. Both of the micrographs revealed that the interface of the interlocked bi-materials was composed of metal (monomodal or bimodal SS316L) and ceramic (3YSZ) powders. According to Figure 7b,c, the monomodal and bimodal SS316L powder particles were moderately coated with the binder system, whereas the 3YSZ powder particles were entirely coated.

### 3.5. Extraction of Binders

**Figure 8:**
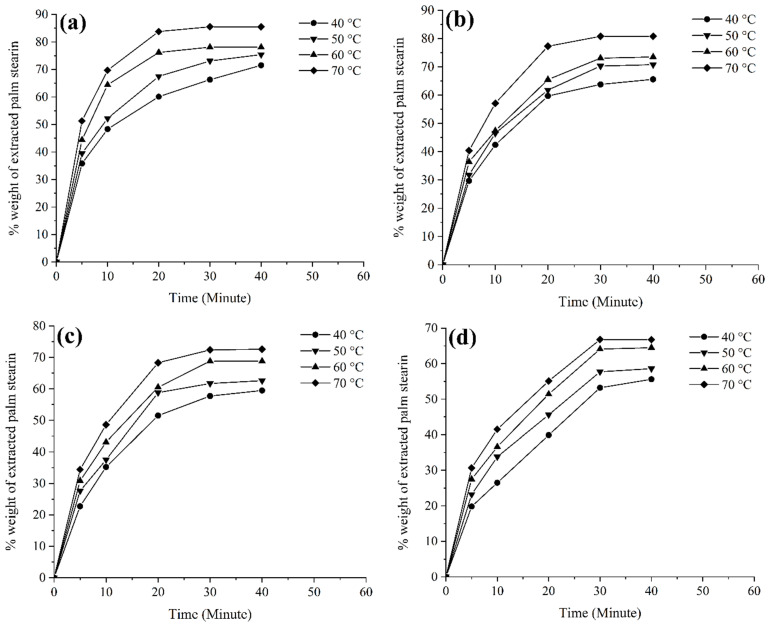
(**a**) Mass loss of palm stearin binder during solvent extraction process from (**a**) monomodal SS316L/3YSZ, (**b**) 15:85 bimodal SS316L/3YSZ, (**c**) 30:70 bimodal SS316L/3YSZ, and (**d**) 45:55 bimodal SS316L/3YSZ micro-components.

Microchannels are usually developed during the solvent debinding process because of the dissolution of binders in a solvent. These microchannels serve as vapour pathways to aid in the extraction of the remaining binders during the thermal debinding process [32]. Such a two-stage debinding procedure was adopted in this investigation. Figure 8 displays the amount of palm stearin extracted over time at different temperatures from the green bi-materials. As the solvent debinding temperature and time increased, Figure 8 shows that the mass loss of palm stearin in bi-materials increased as well, in line with previous research works [29,54]. In the current research, increasing the temperature from 40 °C to 70 °C significantly increased the rate of palm stearin elimination in monomodal SS316L/3YSZ, 15:85 bimodal SS316L/3YSZ, 30:70 bimodal SS316L/3YSZ, and 45:55 bimodal SS316L/3YSZ micro-components from 71.5%, 65.6%, 59.5%, and 55.6% to 85.5%, 80.8%, 72.6%, and 66.8%, respectively. The regulation of the solvent extraction procedure was largely dependent on the diffusion mechanism. Temperature was a major determinant of the diffusion rate; hence, higher temperatures led to more binder elimination as a consequence of the higher diffusion rate [55,56]. For this experiment, the solvent debinding temperature of 70 °C was set and a substantial quantity of palm stearin was removed at this temperature. Temperatures above 70 °C typically contribute to a faster withdrawal rate of the soluble binder and the softer backbone polymer, which increases the risk of a bi-material joining failure. A comparison of Figure 8a with Figure 8b–d revealed that incorporating nano-sized SS316L powder particles lowered the rate of palm stearin removal at various solvent-debinding temperatures. More precisely, bimodally configured bi-materials displayed 5.5–21.9% less palm stearin removal than monomodally configured bi-materials at 70 °C. This could be attributed to the higher surface areas of SS316L nanoparticles, which increased the interparticle friction in the bimodal SS316L micro-components, resulting in flow restrictions and a slower debinding rate.

**Figure 9:**
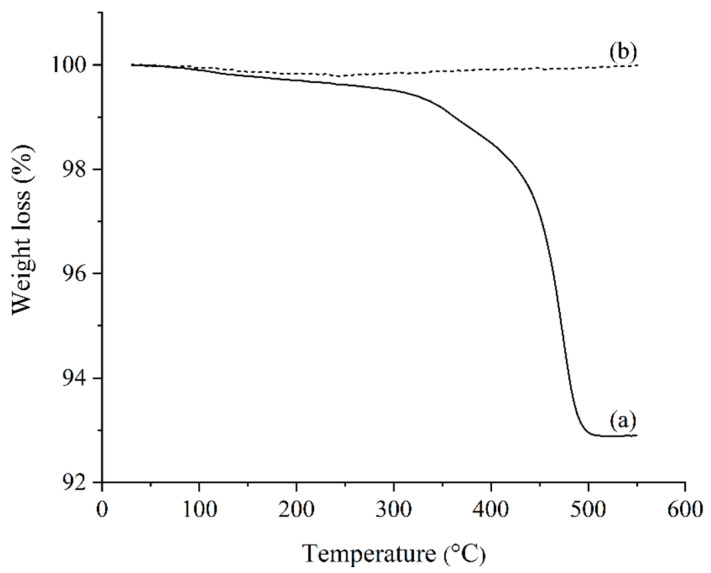
TGA graph of 45:55 bimodal SS316L/3YSZ micro-component (a) before and (b) after thermal debinding at 550 °C.

All of the solvent-debound bi-materials were subjected to very slow heating rates (0.1 °C/min and 0.25 °C/min) during the thermal extraction process to remove the LDPE and the residual palm stearin binders. According to earlier research [57], such a pathway was followed to prevent cracks and other defects from forming in bi-materials. The TGA curves, as shown in Figure 9, reveal the complete removal of the binder system from the bi-materials after the thermal debinding process.

### 3.6. Properties of Sintered Bi-Materials

**Figure 10:**
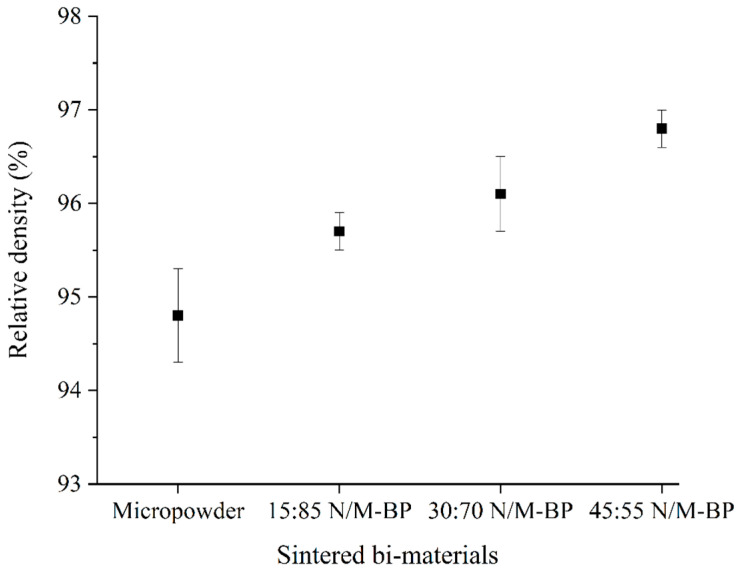
Variation in relative densities in sintered bi-materials with increasing SS316L nanoparticle contents.

Component densification and shrinkage are caused by sintering, which removes the pore spaces between powder particles. The relative density determination of the sintered samples is considered a useful technique to evaluate the sintering process. The relative densities of the bi-materials sintered at 1350 °C for 3 h are illustrated in Figure 10. According to Figure 10, the bimodal SS316L/3YSZ micro-components exhibited higher relative densities than the monomodal SS316L/3YSZ micro-components. The dense grain boundary of the SS316L nanoparticles could be responsible for increasing the sintered density of the bimodal SS316L/3YSZ micro-components [58]. The dense grain boundary developed around the SS316L microparticles when the SS316L nanoparticles, which possessed large surface energy, were sintered prior to the SS316L microparticles. As the grain boundary is a favourable diffusion channel, activated grain boundary diffusion led to an improvement in the densification process [32,59]. As a result, the N/M-BP bi-materials exhibited greater relative densities than the micropowder bi-materials, while the highest relative density of 96.8% was obtained for the 45:55 bimodal SS316L/3YSZ micro-components with an SS316L nanoparticle content of 45 vol.%.

**Figure 11:**
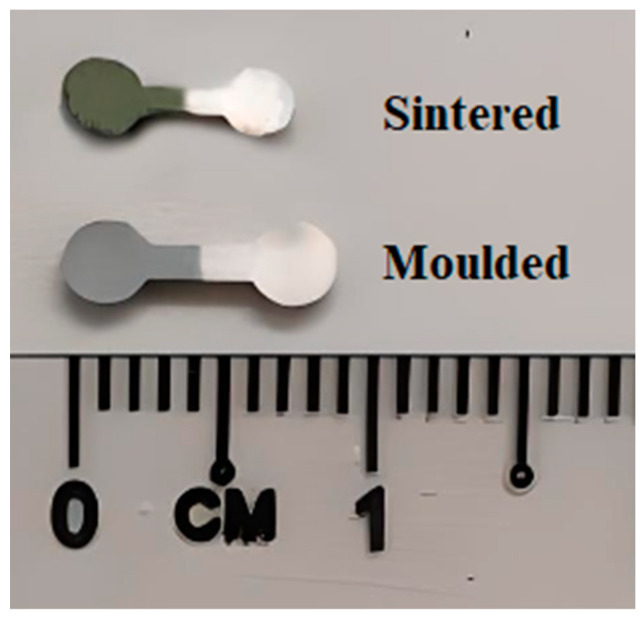
Photograph of micro-injection moulded and sintered bi-material.

**Figure 12:**
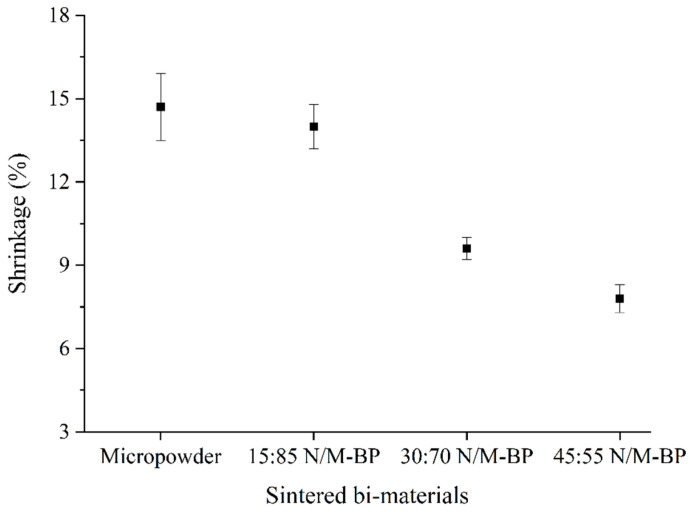
Variation in linear shrinkages in sintered bi-materials with increasing SS316L nanoparticle contents.

All of the 2C-µPIM-processed sintered bi-materials displayed linear shrinkage, as illustrated in Figure 11. As shown in Figure 12, the monomodal SS316L/3YSZ micro-components exhibited the maximum linear shrinkage of 14.7%; however, the shrinkage values in the bi-materials began to decrease and reached 14% when the 15 vol.% nano-sized SS316L powder was added to the micro-sized SS316L powder. While the shrinkage difference between the monomodal SS316L/3YSZ micro-components and the 15:85 bimodal SS316L/3YSZ micro-components was very small, when the 30 vol.% nano-sized SS316L powder was added to the micro-sized SS316L powder instead of the 15 vol.% nano-sized SS316L powder, the shrinkage dropped dramatically. In this investigation, the lowest shrinkage was obtained for the 45:55 bimodal SS316L/3YSZ micro-components, which was 7.8%, representing a 47% drop over the bi-materials consisting solely of the micro-sized SS316L powder. It has been reported that a shrinkage of 15% to 25% is typical for micro-injection-moulded components [60,61]. When such earlier studies are compared with the current investigation, it becomes evident that the N/M-BPs of SS316L contribute to the increased densification in the bimodal SS316L/3YSZ micro-components with relatively low shrinkage. The lesser shrinkage perceived in the bimodal SS316L/3YSZ bi-materials can be attributed to the higher power loadings than those in the monomodal SS316L/3YSZ bi-materials.

**Figure 13:**
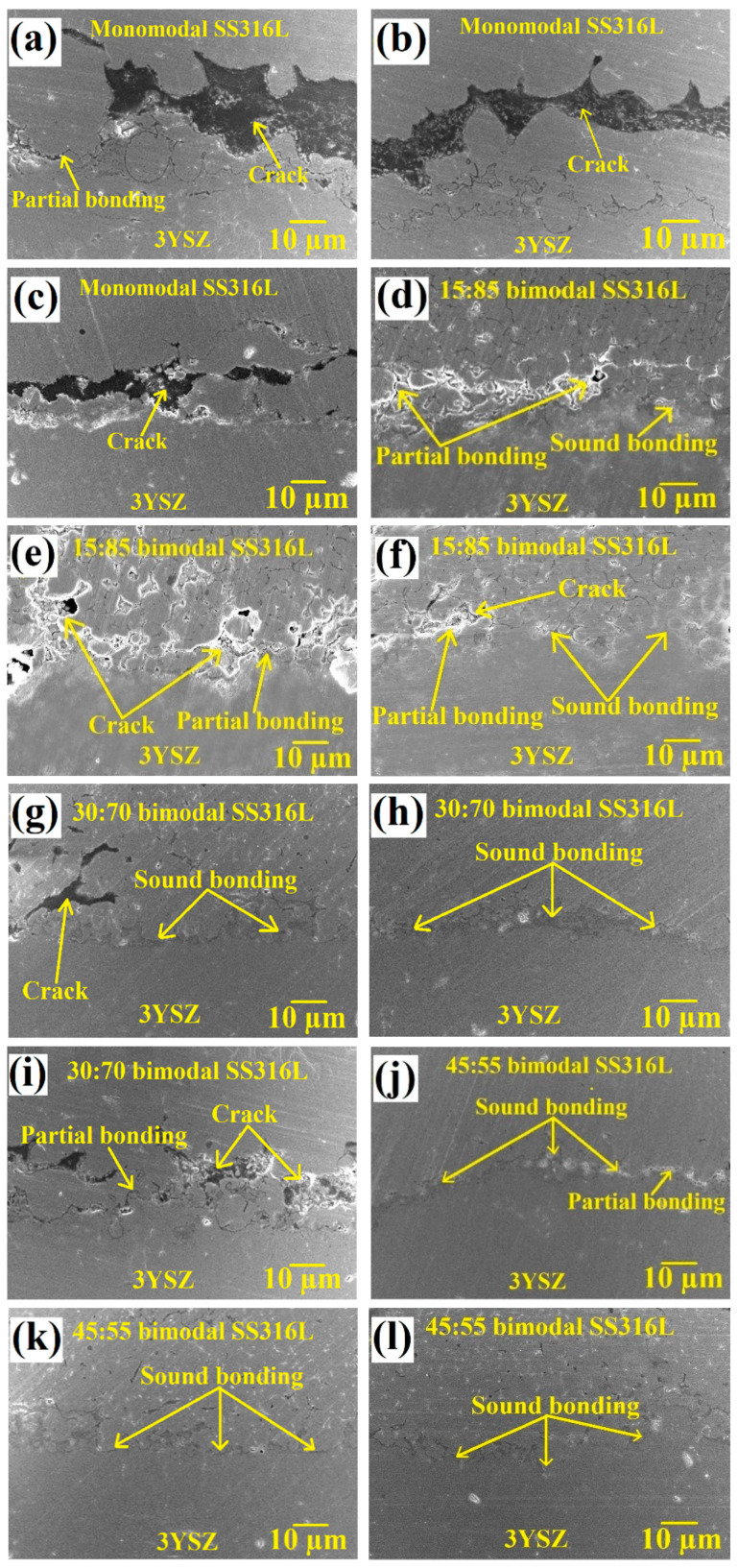
FESEM images exhibiting three different regions of the interfaces of the bi-materials: (**a**–**c**) monomodal SS316L/3YSZ micro-component, (**d**–**f**) 15:85 bimodal SS316L/3YSZ micro-component, (**g**–**i**) 30:70 bimodal SS316L/3YSZ micro-component, and (**j**–**l**) 45:55 bimodal SS316L/3YSZ micro-component.

**Figure 14:**
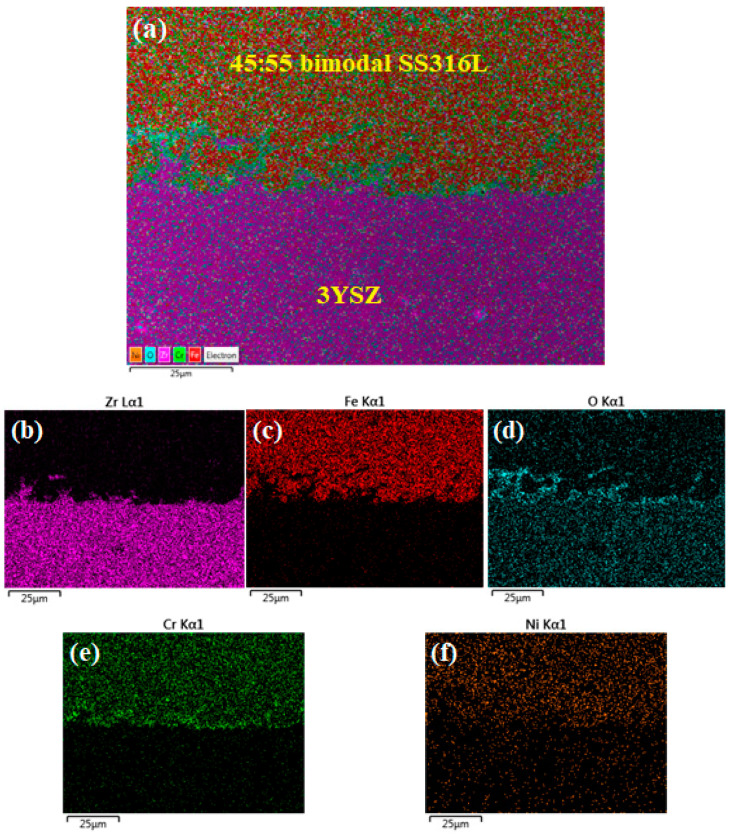
EDX mapping of the sintered 45:55 bimodal SS316L/3YSZ micro-component: (**a**) layered image, (**b**) Zr map, (**c**) Fe map, (**d**) O map, (**e**) Cr map, and (**f**) Ni map.

The FESEM images of the sintered bi-materials are illustrated in Figure 13. To appropriately comprehend the metal/ceramic joining scenario of the monomodal SS316L/3YSZ and bimodal SS316L/3YSZ micro-components, FESEM images were taken at three distinct locations of the interfaces of the samples. According to Figure 13a–c, for the monomodal SS316L/3YSZ micro-component, while a partially bonded interface with cracks was found in one location, the other locations revealed just cracks with no discernible joined interface. Usually, two distinct pieces shrink at varying rates during the sintering process, which causes biaxial mismatch stresses to arise at the region of contact and could contribute to interface cracking [62]. The massive cracks observed in the monomodal bi-materials significantly reduced when the SS316L nanoparticles were added to the SS316L microparticles. According to Figure 13d–f, for the 15:85 bimodal SS316L/3YSZ micro-component, in addition to the comparatively small-sized crack development, partial bonding between the metal and the ceramic particles occurred at different locations of the bi-material, while some strong bonding was also perceived. The metal/ceramic interface of the bi-material was significantly improved by the addition of 30 vol.% of the SS316L nanoparticles, up from the 15 vol.% previously. In the case of the 30:70 bimodal SS316L/3YSZ micro-component, as shown in Figure 13g–i, while sound bonding between bimodal SS316L and 3YSZ was observed at multiple locations of the bi-material, there was still the existence of partial bonding and cracks. In comparison to the 15:85 bimodal SS316L/3YSZ bi-material, significantly less partial bonding was identified in the 30:70 bimodal SS316L/3YSZ bi-material. In this study, cracks were eliminated entirely from different locations of the bi-material interface when the 45-vol.% SS316L nanoparticles were added (Figure 13j–l). As demonstrated in Figure 13j–l, the 45:55 bimodal SS316L/3YSZ bi-material exhibited a crack-free interface as well as strong metal/ceramic bonding, with insignificant partial bonding found at a certain location. The significant reduction in cracks in the 15:85 bimodal SS316L/3YSZ and 30:70 bimodal SS316L/3YSZ bi-materials as well as the complete eradication of cracks in the 45:55 bimodal SS316L/3YSZ bi-material could be attributed to the higher power loadings and consequently lesser shrinkages than those in the monomodal SS316L/3YSZ bi-material. Higher powder loading in bi-materials improves bonding between two different materials by increasing packing density and surface contact at the interface. During the sintering process, higher powder loading allows for improved particle diffusion at the interface, leading to stronger inter-material bonding. In addition, increased powder loading reduces the likelihood of voids, which can compromise total structural integrity. The EDX mapping of the 45:55 bimodal SS316L/3YSZ micro-component is shown in Figure 14. According to this figure, zirconium (Zr), iron (Fe), oxygen (O), chromium (Cr), and nickel (Ni) existed at the interface of the sintered bi-material. The bonding of 45:55 bimodal SS316L and 3YSZ was established via the inter-diffusion of the elements at the interface as a consequence of their higher propensity for oxygen in 3YSZ, leading to the formation of an oxide layer.

**Figure 15:**
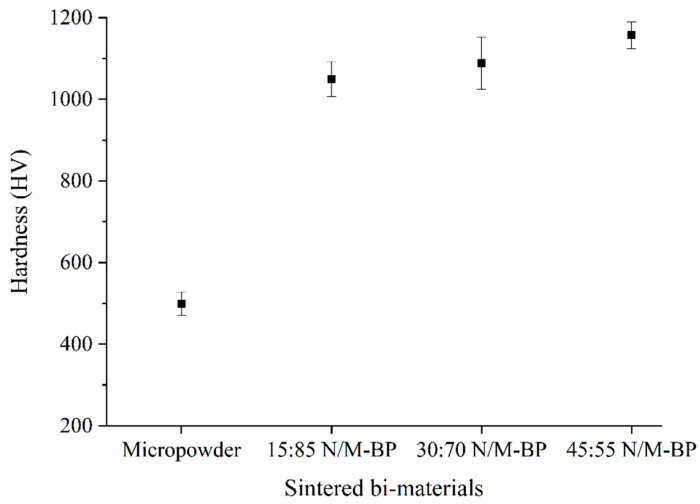
Effect of addition of nanoparticles on the hardness values of the joining region of sintered bi-materials.

Figure 15 demonstrates the values of hardness at the joining region of the sintered bi-materials. According to Figure 15, the inclusion of 15 vol.% SS316L nanoparticles resulted in a dramatic increase in hardness, from 498.4 HV to 1049.4 HV. The development of massive cracks at different locations of the bi-material interface caused the monomodal SS316L/3YSZ micro-components to exhibit significantly low hardness at the joining region. When the 30-vol.% SS316L nanoparticles were added, and the hardness value of the 30:70 bimodal/3YSZ micro-components increased by approximately 3.8% as compared to the 15:85 bimodal/3YSZ micro-components. The highest hardness value of 1156.8 was measured at the joining region of 45:55 bimodal/3YSZ micro-components, which was approximately 2.3 times higher than that observed in the case of the monomodal SS316L/3YSZ micro-components. The increased density, along with the progressive reduction and eventual total removal of cracks from the joining region, might contribute to the improved hardness of the N/M-BP bi-materials.

## 4. Conclusions

The influence of SS316L nanoparticles on the metal/ceramic joining as well as the final sintered properties of the micro-sized bi-materials processed through 2C-μPIM was investigated. On the basis of the above-mentioned experimental results and discussions, the following conclusions were drawn:

- When compared to the SS316L micropowder, the exhibition of a 9.04–19.45% increase in the critical powder loadings in the nano/micro-bimodal SS316L powders (with nanoparticle contents ranging from 15 vol.% to 45 vol.%) signified that the bimodally configured nano/micro distributions were preferred for improving the powder loading because of the capacity of the nanoparticles to occupy the interstitial gaps within the microparticles;
- The rheological analysis of the monomodal SS316L, bimodal SS316L, and 3YSZ feedstocks demonstrated pseudo-plastic behaviour. The viscosity of all of the feedstocks dropped with increasing temperatures. The viscosity of the bimodal SS316L feedstocks with different SS316L nanoparticle contents was higher than that of the monomodal SS316L feedstock, implying that the large specific surface area of the nanoparticles led to higher interparticle friction and elevated viscosities in the bimodal feedstocks;
- Following sintering, the N/M-BP bi-materials exhibited greater relative densities than the micropowder bi-materials; the 45:55 bimodal SS316L/3YSZ micro-components yielded the highest relative density of 96.8%. The sintered micropowder bi-materials had the highest linear shrinkage of 14.7%, while the shrinkage values in the bi-materials lowered to 7.8% when the 45 vol.% SS316L nanoparticles were added to the SS316L microparticles. The evaluation of the microstructures revealed that the addition of the SS316L nanoparticles not only dramatically reduced the generation of massive cracks as observed in the micropowder bi-materials but also improved the metal/ceramic bonding in the N/M-BP bi-materials eventually;
- The joining region of the bimodally configured sintered bi-materials with an SS316L nanoparticle content of 45 vol.% demonstrated the greatest hardness value of 1156.8, which was almost 2.3 times that of the monomodal bi-materials. A potential future direction for this research could involve investigating the influence of different sintering environments on the sintered properties and long-term reliability of the bi-materials.

## Figures and Tables

**Table 1:** Characteristics and properties of palm stearin and LDPE binders.

Binders	Chemical Structure	Content (wt.%)	Melting Point (°C)	Decomposition Range (°C)	Tensile Strength (kgf/cm^2^)	Tensile Elongation (%)
Palm stearin	CH_3_(CH_2_)_14_COOH	60	57.6	340.5–460.6	–	–
LDPE	(C_2_H_4_)*n*	40	110.2	385.5–505.3	110	400

**Table 2:** Micro-injection parameters to produce green metal/ceramic micro-components.

Melt Temperature (°C)	Mould Temperature (°C)	Injection Pressure (bar)	Injection Time (s)
230	140	12	6

**Table 3:** Flow behaviour index at various temperatures for bimodal SS 316L and 3YSZ feedstocks.

Feedstocks	Temperature (°C)	Flow Behaviour Index (*n*)
Monomodal SS 316L	190	0.546
	210	0.397
	230	0.384
15:85 bimodal SS 316L	190	0.574
	210	0.468
	230	0.394
30:70 bimodal SS 316L	190	0.656
	210	0.518
	230	0.420
45:55 bimodal SS 316L	190	0.685
	210	0.528
	230	0.503
3YSZ	190	0.713
	210	0.558
	230	0.474

**Table 4:** Flow activation energy (*E*) of the feedstocks.

Feedstocks	Flow Activation Energy (KJ/mol)
Monomodal SS316L	11.28
15:85 bimodal SS316L	19.54
30:70 bimodal SS316L	20.79
45:55 bimodal SS316L	24.94
3YSZ	12.47

## Data Availability

The original contributions presented in the study are included in the article, further inquiries can be directed to the corresponding author.
